# Anti-angiogenic and antitumor activities of Huaier aqueous extract

**DOI:** 10.3892/or.2012.1961

**Published:** 2012-08-08

**Authors:** XIAOLONG WANG, NING ZHANG, QIANG HUO, QIFENG YANG

**Affiliations:** Department of Breast Surgery, Qilu Hospital, Shandong Univeristy School of Medicine, Jinan, Shandong 250012, P.R. China

**Keywords:** Huaier, angiogenesis, tumor, 4T1, human umbilical vein endothelial cell

## Abstract

Traditional Chinese medicine, a rich source of potent cancer chemopreventive agents, is attracting increasing attention worldwide. Recently, the anticancer activity of *Trametes robiniophila* Murr. (Huaier) has been widely investigated. However, the mechanisms are not yet fully understood. This study aimed to elucidate the inhibitory effect of Huaier extract on angiogenesis and tumor growth. Incubation with Huaier extract inhibited the proliferation of human umbilical vein endothelial cells (HUVECs) and mouse mammary tumor cells (4T1). In addition, treatment with Huaier extract decreased the motility and tube formation of HUVECs in a dose-dependent manner *in vitro*. As determined by western blot analysis, Huaier extract dose-dependently decreased the levels of phosphorylated extracellular signal-regulated kinase (ERK), transcription factor p65, c-Jun N-terminal kinase (JNK), signal transducer and activator of transcription 3 (STAT3) and the expression of vascular endothelial growth factor (VEGF). In *ex vivo* experiments, new vessel growth was suppressed as shown by chick embryo chorioallantoic membrane (CAM) and rat aortic ring assays in the presence of Huaier extract. To further evaluate the inhibitory effect, 4T1 cells were injected subcutaneously into BALB/c mice. The administration of Huaier extract suppressed tumor volume, decreased microvessel density and induced apoptosis. These data suggest that Huaier extract may serve as a potent anti-angiogenic and antitumor agent.

## Introduction

Cancer, a multi-step and systematic disease, is a leading cause of mortality worldwide. In 2008, 7.6 million deaths due to cancer were recorded (approximately 13% of the total number of deaths) (1). As one of the main types of cancer, breast cancer accounted for 458,000 deaths in 2008 (1). There were almost 230,480 new cases of invasive breast cancer and 39,520 breast cancer deaths among women in the US 2011 (2). In developing countries, breast cancer occupies approximately half of the total number of breast cancer cases worldwide and 60% of deaths, and is the leading cause of cancer mortality among women in this area (3). In China, the incidence of breast cancer increased rapidly from 126,227 cases in 2002 (4) to 169,452 in 2008 (5). However, currently available chemotherapy treatments provide little benefit, combined with serious side-effects and dose-limiting toxicities (6). Therefore, agents which can interfere with the essential steps of cancer development, such as angiogenesis, are being increasingly used in the treatment of human cancer (7,8).

Angiogenesis is a complex process, referring to the formation of new blood vessels from pre-existing ones (9). During angiogenesis, several steps are involved: degradation of the extracellular matrix, migration, proliferation, sprouting, elongation and tube formation of endothelial cells (10). It is well known that physiological angiogenesis has a great contribution to embryonic development, wound healing and tissue regeneration (11–13), and it is tightly controlled by the balance between the pro-angiogenic factors, such as vascular endothelial cell growth factor (VEGF) and anti-angiogenic factors, such as endostatin. Forming new blood vessels is an essential step in tumor development (14–17). Without blood supply, the tumor volume will not exceed 1–2 mm^3^(18). Tumor development can only continue with the formation of new blood vessels (19). Therefore, anti-angiogenesis is a promising strategy for cancer treatment.

Since angiostatin and endostatin were recognized as endogenous anti-angiogenic factors, a number of phytochemicals, such as *Salvia officinalis*(20), cinnamon extract (21), and koetjapic acid from *Sandoricum koetjaoe* Merr. (22), have been proven to possess anti-angiogenesis activities. *Trametes robiniophila* Murr. (Huaier extract) a traditional Chinese medicine (TCM), has been widely used in China for many years. Previous studies have reported that Huaier extract inhibits the growth of hepatocellular carcinoma cells (23,24), and our previous study showed that Huaier aqueous extract inhibited the proliferation of breast cancer cells by inducing apoptosis (25). Although the antitumor activity of Huaier extract has been revealed, the exact underlying mechanisms remain largely unknown. In the present study, we evaluated the anti-angiogenic effect of Huaier extract, in combination with its antitumor effects.

## Materials and methods

### Reagents

The human umbilical vein endothelial cell line (HUVEC) and mouse mammary tumor cell line, 4T1, were purchased from the American Type Culture Collection (ATCC, Manassas, VA, USA), and were routinely cultured in DMEM medium (Gibco-BRL, Rockville, IN, USA) containing 10% FBS (Haoyang Biological Manufacturer Co., Ltd., Tianjin, China), 100 U/ml penicillin and 100 μg/ml streptomycin in 5% CO_2_ at 37°C. Anti-p21, anti-extracellular signal-regulated kinase (ERK), anti-phosphorylated (p)-ERK, anti-c-Jun N-terminal kinase (JNK), anti-p-JNK, anti-p65, anti-p-p65, anti-signal transducer and activator of transcription 3 (STAT3) and anti-p-STAT3 (ser727) antibodies were obtained from Cell Signaling Technology, (Beverly, MA, USA). Anti-VEGF antibody was provided by Abcam, (Cambridge, MA, USA). Anti-β-actin (1:5000) antibody was obtained from Sigma-Aldrich (St. Louis, MO, USA). Anti-mouse and rabbit IgG horseradish peroxidase (HRP) antibodies (1:5000) was from ZhongShan Goldenbridge Biotechnology Co., Ltd. (Beijing, China). The pro-lighting HRP agent for western blot analysis was supplied by Tiangen Biotech Co., Ltd., (Beijing, China).

### Preparation of Huaier aqueous extract

Electuary ointment of Huaier extract was kindly provided by Gaitianli Medicine Co., Ltd. (Jiangsu, China). The electuary ointment (2 g) was soaked in 20 ml of DMEM. The solid residue of the above dissolved herbs was filtered and discarded through a 0.22-μm filter. The final 100 mg/ml stock solution was kept at −20°C for long storage.

### Effect of Huaier extract on cell morphology

HUVECs were seeded in a 24-well plate. After 12 h, the HUVECs were exposed to various concentrations of Huaier extract for an additional 24 h. Finally, the morphological changes caused by Huaier extract were observed under an Olympus light microscope and photomicrographs were taken with an Olympus digital camera (Olympus, Tokyo, Japan).

### 3-(4,5-Dimethylthiazol-2-yl)-2,5-diphenyltetrazolium bromide (MTT) assay

MTT assay was performed to measure the viability of the HUVECs and 4T1 cells after treatment with Huaier extract. In brief, the HUVECs (10^3^ cells/well) and 4T1 cells (700 cells/well) were seeded in cultured medium in 96-well plates and incubated in 5% CO_2_ at 37°C. After 12 h, the medium in each well was replaced with the vehicle or different concentrations (2, 4 and 8 mg/ml) of Huaier extract and incubated for another 48 or 72 h. Subsequently, 20 μl of MTT (5 mg/ml in PBS) were added into each well. After 4 h of incubation at 37°C, the supernatants were aspirated carefully and 100 μl of dimethyl sulfoxide (DMSO) were added to each well. Absorbance values at 490 nm were determined by the Microplate Reader (Bio-Rad, Hercules, CA, USA).

### Cell cycle analysis

After 24 h of starvation in serum-free medium at 37°C, the HUVECs were treated with various concentrations of Huaier extract or complete medium as the negative control. After 24 h, the treated cells were harvested, washed with 1X cold PBS and fixed with 70% ice-cold ethanol overnight. After the ethanol was removed by centrifugation at 1,200 × g for 1 min, the fixed cells were washed with PBS twice. The pellets were then resuspended with 1 ml of DNA staining solution (MultiSciences Biotech Co., Ltd.). After incubation for 30 min at room temperature in the dark, cells were analyzed in the presence of the dye by FACScan flow cytometry (Becton-Dickinson, Franklin Lakes, NJ, USA) and the data were analyzed by ModFitLT V2.0 software (Becton-Dickinson).

### Propidium iodide (PI)-Annexin-V staining analysis

The BD Pharmingen™ PE Annexin V Apoptosis Detection Kit (BD Biosciences, Franklin Lakes, NJ, USA) was used to detect the proportion of apoptotic cells, according to the instructions of the manufacturer. Briefly, after treatment with various concentrations of Huaier extract for the indicated times, the HUVECs were harvested and washed with PBS twice. The cells (1×10^5^) were then resuspended in 100 μl of binding buffer, followed by adding 5 μl Annexin V-FITC and 5 μl PI. After incubation for 15 min in the dark, another 400 μl of binding buffer were added before the cells were analyzed by FACScan flow cytometry.

### In vitro scratch assay

Scratch assay was applied to determine cell mortality caused by Huaier extract. This assay was performed using a standard method (26) with some modifications. Briefly, 2.5×10^4^ HUVECs were seeded on a 12-well plate in complete medium overnight to obtain a full confluent monolayer. After 24 h of starvation, a 20-μl pipette tip was used to create a straight cell-free wound. Each well was washed twice with PBS to remove debris. The cells were then cultured in serum-free medium in the absence or presence of various concentrations of Huaier extract. The distances between the 2 edges of the scratch were analyzed quantitatively.

### Cell migration assay

*In vitro* cell migration assay was performed using the Transwell system (24-wells, 8-μm pore size with polycarbonate membrane; Corning Costar, Lowell, MA, USA). Cells were starved in serum-free medium for 24 h at 37°C. The HUVECs were then harvested and resuspended in various concentrations of Huaier extract diluted in serum-free medium. The conditional medium from the NIH3T3 fibroblasts and the complete medium were then mixed (v/v 1:1). Subsequently, 1 ml of the mixture was added to the lower well of each chamber, and 100 μl of cell solutions containing 1×10^4^ HUVECs were added to the upper wells. After treatment for 24 h, the cells attached to the lower surface were fixed with methanol and stained with 0.2% Giemsa. The successfully migrated cells were counted on 5 random fields using an Olympus light microscope.

### Tube formation assay

The ability of the HUVECs to form network structures was tested on Matrigel basement membrane matrix (BD Biosciences, San Jose, CA, USA). Firstly, 50 μl of Matrigel were plated per well on 96-well plates and allowed to polymerize at 37°C for 30 min. Subsequently, 100 μl of HUVECs suspended in complete medium at a density of 1×10^5^/ml were added to each well in the absence or presence of Huaier extract. After 9 h, tube-like structures were photographed with an Olympus digital camera.

### Chick embryo chorioallantoic membrane (CAM) assay

CAM assay was performed as described previously (27) with small modifications. Briefly, 40 fertilized chicken eggs were incubated at 37°C at constant humidity and randomly divided into 4 groups. On the 9th day of incubation, a square window (1×1 cm^2^) was opened in the shell. The following day, filter discs loaded with 20 μl complete medium or various concentrations of Huaier extract were placed on the top of the growing CAMs under sterile conditions. Afterwards, the window was sealed with sterilized surgical tape and the eggs were returned to the incubator. After 24 h of incubation, the CAMs were photographed using an Olympus Live View Digital SLR camera.

### Rat aortic ring assay

Angiogenesis *ex vivo* was also studied by rat aortic ring assay (28). Briefly, a 48-well plate was first covered with Matrigel and incubated for 30 min at 37°C. Subsequently, 2-month old BALB/c mice were sacrificed by cervical dislocation, and the thoracic aortas were dissected and cut into 1–2-mm long sections. Afterwards, aortic rings were placed into wells pre-coated with Matrigel, and then covered with another layer of Matrigel. After 30 min of polymerization, DMEM supplemented with 20% FBS was added into each well. The following day, the supernatants were replaced with medium in the absence or presence of various concentrations of Huaier extract. On day 6, the fields covered by the sprouting from the aortic rings were measured by an Olympus digital camera.

### Western blot analysis

In brief, the HUVECs were allowed to grow to 60–70% confluence in 25 cm^2^ cell culture flasks, and then incubated with gradient concentrations of Huaier extract at 37°C under 5% CO_2_. After 2 h of treatment, the cells were harvested, and the proteins were lysed in lysis buffer (1X PBS, 1% NP40, 0.1% sodium dodecyl sulfate, 5 mM EDTA, 0.5% sodium deoxycholate and 1 mM sodium orthovanadate) with protease inhibitors. Subsequently, 50 μg of total cellular protein from each sample were separated by 10% SDS-PAGE and electrotransferred onto a polyvinylidene fluoride (PVDF) membranes by using a semi-dry blotting apparatus (Bio-Rad). After blocking with 5% non-fat milk, the PVDF membranes were covered with specific primary antibodies, followed by incubation with secondary antibodies. The protein bands were then visualized by using Pro-lighting HRP agent and their densities were analyzed using ImageJ software. β-actin was used as the loading control.

### Animals and tumor model

Twenty BALB/c female mice, 4–5 weeks old, were purchased from the Center for New Drugs Evaluation of Shandong University, and housed under pathogen-free conditions. All the experiments were approved by the institutional guidelines of the Animal Care and Use Committee at Shandong University. 4T1 cells (1×10^6^) were subcutaneously injected into the left flank of each mouse. After 2 days, each mouse was given 100 μl solution containing 50 mg Huaier extract by gavage daily. After 21 days, the mice were sacrificed, and the xenografts were removed for immunohistochemical staining.

### Histology and immunohistochemistry

Immediately after excision, the tumor tissues were stored in 10% neutral-buffered formalin. After 24 h, the samples were paraffin-embedded and then sliced into 4-μm section for hematoxylin and eosin (H&E) staining according to the standard techniques. The relative areas of necrosis in tumors were analyzed.

To quantify the microvessel density (MVD), the SP-9000 Histostain™-Plus Kits (ZhongShan Goldenbridge Biotechnology Co.) were used to detect CD34 expression using the standard steps. Briefly, the sections were deparaffinized and rehydrated, followed by antigen retrieval with pH 6.0 citrate buffer. Endogenous peroxidase activity was inhibited with 3% H_2_O_2_ for 15 min and the sections were incubated with 10% normal goat serum to block non-specific binding. After incubation with anti-CD34 antibody (Santa Cruz Biotechnology, Santa Cruz, CA, USA) at 4°C overnight, the sections were washed, treated with biotinylated anti-immunoglobulin antibody for 20 min and reacted with horseradish peroxidase-conjugated streptavidin. Then the liquid DAB substrate/chromogen system (Maixin Bio, Fuzhou, China) was used, followed by counterstaining with hematoxylin. The representative images of tumor tissues were taken by an Olympus light microscope.

### Terminal deoxynucleotidyl transferase (TdT)-mediated dUTP nick end-labeling (TUNEL) assay

TUNEL assay was performed to identify the apoptotic cells in the paraffin-embedded sections using the One Step TUNEL Apoptosis Assay kit (Beoytime, Beijing, China) according to the manufacturer’s instructions. TUNEL-positive cells were visualized with red fluorescent staining observed by a fluorescence microscope (Olympus).

### Statistical analysis

The results are presented as means ± standard deviation (SD) and differences between groups were compared by one-way ANOVA and considered significant at P<0.05. The statistical analysis was carried out by using SSPS edition 16.0.

## Results

### Effects of Huaier extract on cell morphology and viability of HUVECs

**Figure 1:**
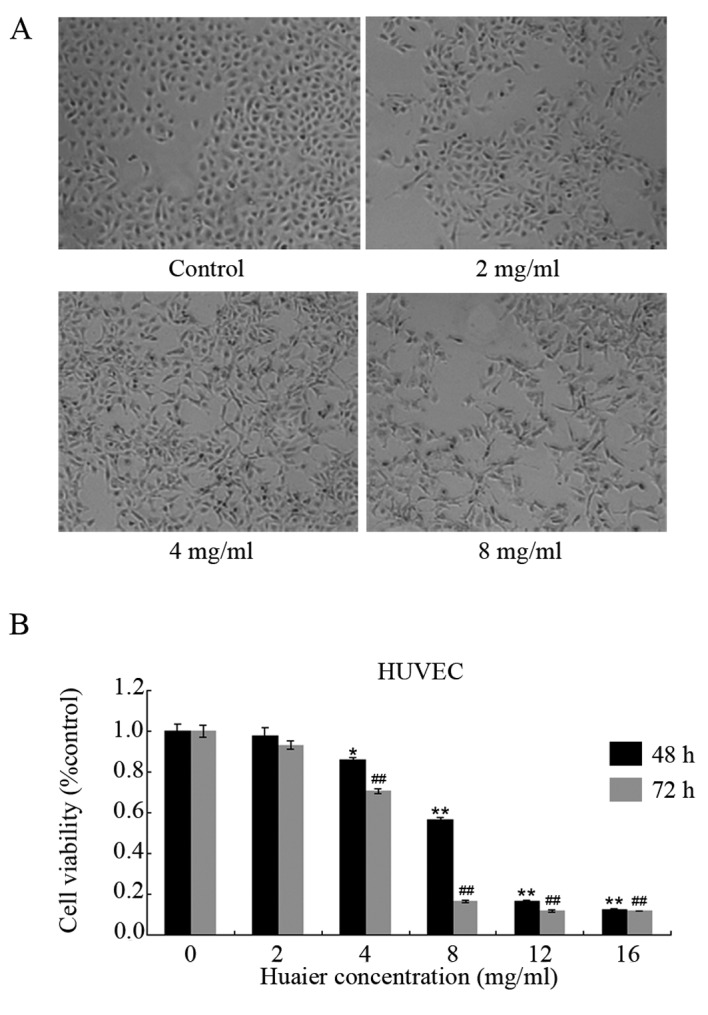
Morphological and viability changes in HUVECs induced by Huaier extract. (A) HUVECs were seeded in 24-well plates and incubated with Huaier extract for 24 h. Representative images of HUVECs in the control and treated groups. (B) Huaier extract inhibited cell proliferation in a time- and dose-dependent manner. The results are presented as the means ± SD of 3 independent experiments conducted in triplicate. ^*^P<0.05; ^**^P<0.01; ^#^P<0.05; ^##^P<0.01.

To investigate the effect of Huaier extract on angiogenesis, we first observed the cell morphology of the HUVECs after exposure to Huaier extract. Following 24 h of incubation with various concentrations of Huaier extract, the morphological changes in the HUVECs were observed (Fig. 1A). Obvious morphological changes were observed in the HUVECs. Compared with the untreated cells, the majority of the cells in the Huaier-treated groups became enlongated and star-shaped with sharp outlines. These results suggest that Huaier extract causes cell skeleton rearrangement in HUVECs.

We then examined the cell viability by using MTT assay. As shown in Fig. 1B, Huaier extract suppressed the proliferation of the HUVECs in a time- and dose-dependent manner. The inhibitory rates of Huaier extract varied from 2.3±3.7% to a maximum of 87.7±0.5% after 48-h incubation, with an IC_50_ of 8.1±0.9 mg/ml. After treatment with increasing concentrations of Huaier extract for 72 h, the viabilities of the HUVECs were suppressed by 6.9±2.2, 29.4±0.9, 83.7±0.5, 87.9±4.5 and 88.0±0.2%, respectively. A significant reduction was firstly observed at 4 mg/ml (P<0.05).

### Suppressive effect on HUVEC proliferation correlates with cell cycle arrest and apoptosis induction

**Figure 2:**
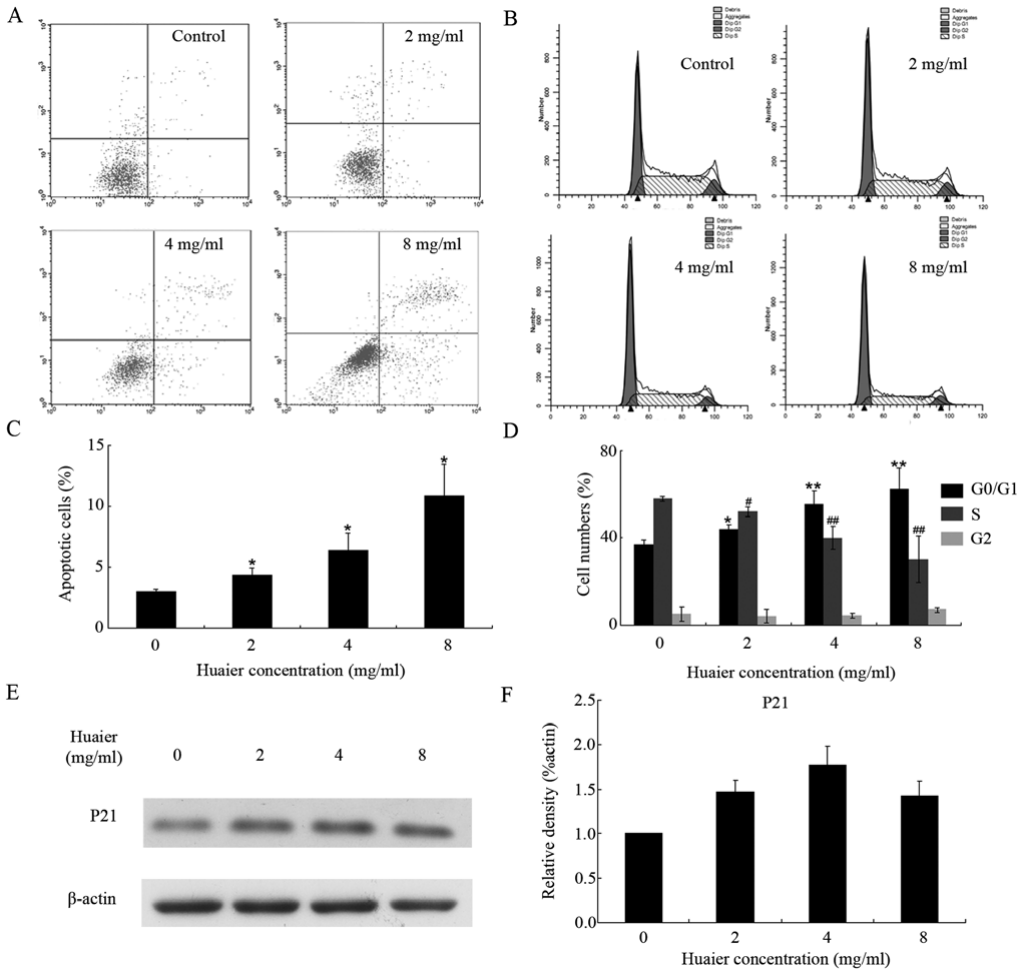
Huaier extract induced apoptosis and caused cell cycle arrest in HUVECs. HUVECs were incubated with increasing concentrations of Huaier extract for up to 48 h. (A and C) Examples of flow cytometric data of HUVECs examined by PI-Annexin-V staining analysis after incubation with Huaier extract for 48 h. (B and D) Representative images of cell cycle analysis are shown, and the percentage of cells in each phase of the cell of each following treatment for 24 h is presented as a histogram, and all values were expressed as the means ± SD of 3 independent experiments conducted in triplicate. (E and F) Huaier extract induced the accumulation of p21. The bar graphs represent the relative density of p21/actin. Con, control. ^*^P<0.05; ^**^P<0.01; ^#^P<0.05; ^##^P<0.01.

To further explore the underlying mechanism of the antiproliferative effect of Huaier extract, we applied flow cytometry to analyze the apoptotic rate and cell cycle distribution after treatment with Huaier extract. The data demonstrated that the ratios of apoptotic HUVECs were 4.4±0.6, 6.4±1.4 and 10.9±2.6% in the presence of increasing concentrations of Huaier extract for 48 h, respectively (Fig. 2A and C). In addition, as shown in Fig. 2B and D, after 24 h of treatment, the proportion of cells at the G0/G1 phase was dose-dependently increased (from 36.79±2.25% in control group to 62.41±9.77% in 8 mg/ml Huaier group). Furthermore, treatment with Huaier extract resulted in the accumulation of p21 with a maximum at 4 mg/ml (Fig. 2E and F), and this partly contributed to the cell cycle arrest mentioned above.

### Effect of Huaier extract on motility of HUVECs

**Figure 3:**
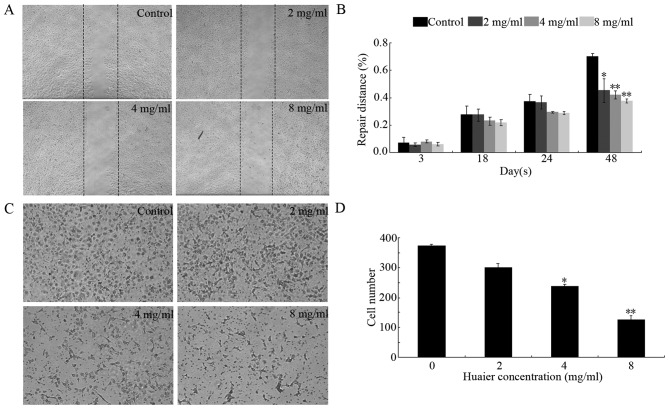
Effect of Huaier extract on the cell migration of HUVECs. (A) Confluent monolayers of HUVECs on a 12-well plate were wounded using a pipette tip and treated with Huaier extract or the vehicle. The images of wound closure were captured under a phase-contrast microscope after 48 h. (B) The migration inhibition is presented as distances between the 2 edges of the scratch. (C) Chemotaxis assay of HUVECs, which shows the inhibitory effect of Huaier extract on cell migration following 24 h of treatment. The cells successfully migrated to the lower surface of the insert. (D) The cell numbers decreased in a dose-dependent manner following treatment with Huaier extract. In the histograms, all values are expressed as the means ± SD. ^*^P<0.05; ^**^P<0.01 as compared with the vehicle.

We then examined the influence of Huaier extract on cell motility by using modified scratch assay (26) and cell migration assay (29). As shown in Fig. 3A and B, after treatment with Huaier extract, the migration of the HUVECs was dose- and time-dependently inhibited. This result was consistent with the results presented in Fig. 3C and D. At 8 mg/ml, the number of cells which had successfully migrated to the lower side of the filter was reduced by 66.2±2.8% (P<0.01) after 24 h of treatment with Huaier extract.

### Effect of Huaier extract on angiogenesis in vitro and ex vivo

**Figure 4:**
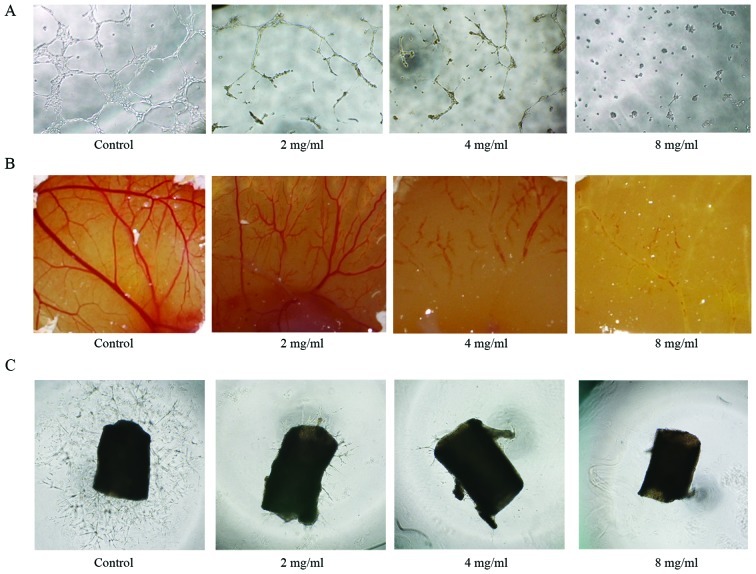
Huaier inhibited angiogenesis *in vitro* and *ex vivo*. (A) HUVECs were seeded on Matrigel-coated 96-well plates and incubated in the absence or presence of Huaier extract for 9 h. Representative images of HUVEC tube formation. (B) CAM of 9-day-old chick embryos exposed to Huaier extract or the vehicle. After 24 h of incubation, the CAM tissue directly beneath each filter disc was photographed. The image represents at least 6 chick embryos. (C) Examples of aortic rings of mice fed with DMEM containing 20% FBS in the control and treated groups. Representative images of vessel sprouting were taken at day 5 of treatment.

As an essential step for angiogenesis, the formation of tube-like structures involves matrix degradation, rearrangement and apoptosis of endothelial cells. As shown in Fig. 4A, untreated HUVECs formed organized capillary tubes within 9 h. While in the presence of Huaier extract, the HUVECs rounded up and rendered incomplete network structures.

To verify the anti-angiogenic effect of Huaier extract *ex vivo*, CAM assay and aortic ring assay were also applied. On day 9 of embryo development, fertilized chick eggs were treated with various concentrations of Huaier extract. After 24 h of incubation, normal vascular pattern with numerous branchings was observed in the control group. However, Huaier extract significantly distorted the vasculature architecture on the chorioallantoic membrane in a dose-dependent manner (Fig. 4B).

The results demonstrated that Huaier extract caused a dramatic decrease in sprout length and density from the aortic ring in a dose-dependent manner (Fig. 4C). In conclusion, Huaier exhibited anti-angiogenic activity both *in vitro* and *ex vivo*.

### Effect of Huaier extract on endothelial signaling pathways

**Figure 5:**
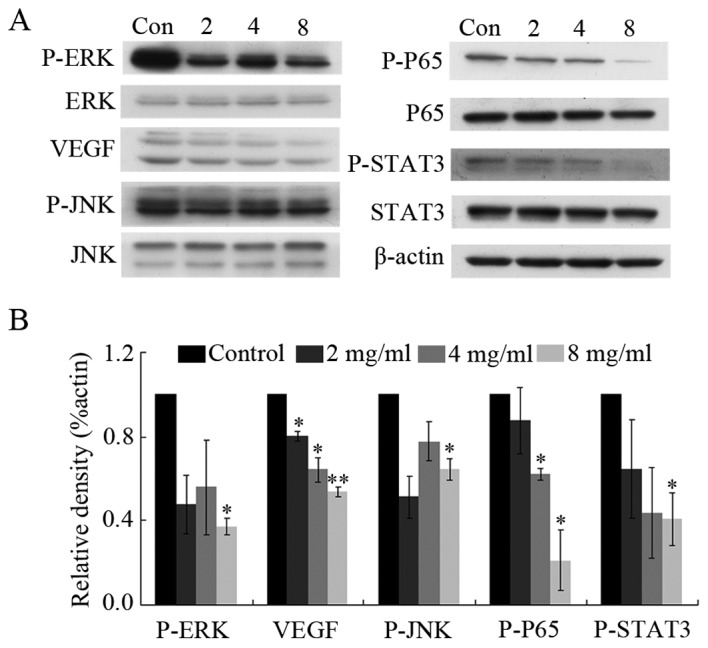
Huaier extract regulated multiple molecules in HUVECs. (A) After HUVECs were treated with various concentrations of Huaier extract, the expression levels of phosphorylated (P)-ERK and ERK were detected after 2 h, while the expression of VEGF was analyzed after 24 h by immunoblotting. β-actin was used as the loading control. (B) The bar graphs represent the mean relative densities ± SD of 3 independent experiments. Con, control. ^*^P<0.05; ^**^P<0.01.

To identify whether Huaier extract can regulate multiple molecules involved in angiogenesis, we used western blot analysis to investigate the changes between the vehicle- and Huaier-treated groups. The results showed that Huaier extract regulated the ERK pathway by down-regulating the phosphorylation of ERK without affecting overall ERK expression levels (Fig. 5). In addition, the expression of VEGF was significantly reduced by 46.4±2.1% after incubation with 8 mg/ml Huaier extract for 24 h (P<0.01). Similarly, Huaier extract reduced the phosphorylation of JNK, STAT3 and p65. However, no effect was observed on Akt signaling and Huaier extract was unable to suppress the level of HIF (data not shown).

### Huaier extract inhibits tumor growth in vitro and in a xenograft model

**Figure 6:**
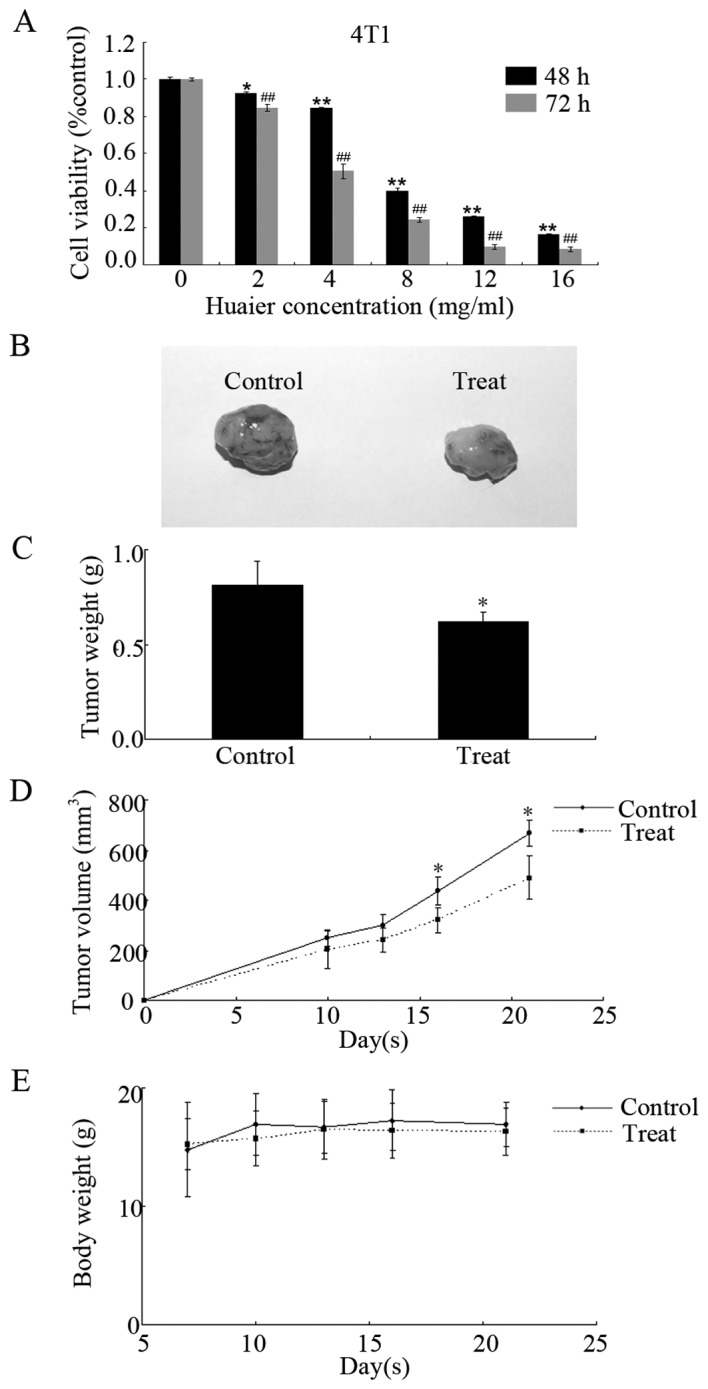
Huaier extract suppressed the growth of 4T1 cells *in vitro* and *in vivo*. (A) Huaier extract inhibited 4T1 cell proliferation as shown by MTT assay. In the *in vivo* experiment, the mice were administrated with Huaier extract by gavage daily for 18 days. (B) Tumor images after mice were sacrificed. (C) Inhibition of tumor growth judging from the final tumor weight. (D and E) Tumor volumes and body weights were measured 7 days later. The bars represent the means ± SD. ^*^P<0.05; ^**^P<0.01; ^#^P<0.05; ^##^P<0.01.

The antiproliferative effect of Huaier extract on 4T1 cells was examined by MTT assay. The results demonstrated that Huaier extract significantly inhibited the proliferation of 4T1 cells in a time- and dose-dependent manner (Fig. 6A). With 2 mg/ml of Huaier extract, a significant suppression on 4T1 cell proliferation was observed with a 7.7±0.9% (at 48 h, P<0.05) and 15.2±1.8% (at 72 h, P<0.01) reduction. The IC_50_ for 4T1 cells was 7.9±0.9 mg/ml (at 48 h) or 4.4±0.6 mg/ml (at 72 h). These data suggest that 4T1 cells are more sensitive to Huaier extract than HUVECs.

Taking into account the anti-angiogenic and antitumor effects of Huaier extract *in vitro* and *ex vivo*, we then examined the antitumor effect of Huaier extract in BALB/c mice. As shown in Fig. 6B and C, the administration of Huaier extract by gavage delayed the tumor volume at concentration of 2.5 g/kg per day. Compared with the control group (667.0±52.6 mm^3^), tumor volumes in the Huaier-treated group were significantly smaller (488.9±86.5 mm^3^, P<0.05) at day 21. The antitumor activity of Huaier extract *in vivo* was confirmed by measuring the tumor weights after the mice were sacrificed. The weights of tumors isolated from the Huaier-treated groups were significantly decreased by 23.6±5.1% (0.81±0.13 g in the control group, and 0.62±0.05 g in the treated group, P<0.05). However, we observed no significant difference between the 2 groups in body weight, which indicated no obvious toxicity to mice at the curative dose (Fig. 6D). Our data prove the antitumor effect of Huaier extract on 4T1 mouse mammary cancer without causing marked toxicity *in vivo*.

**Figure 7:**
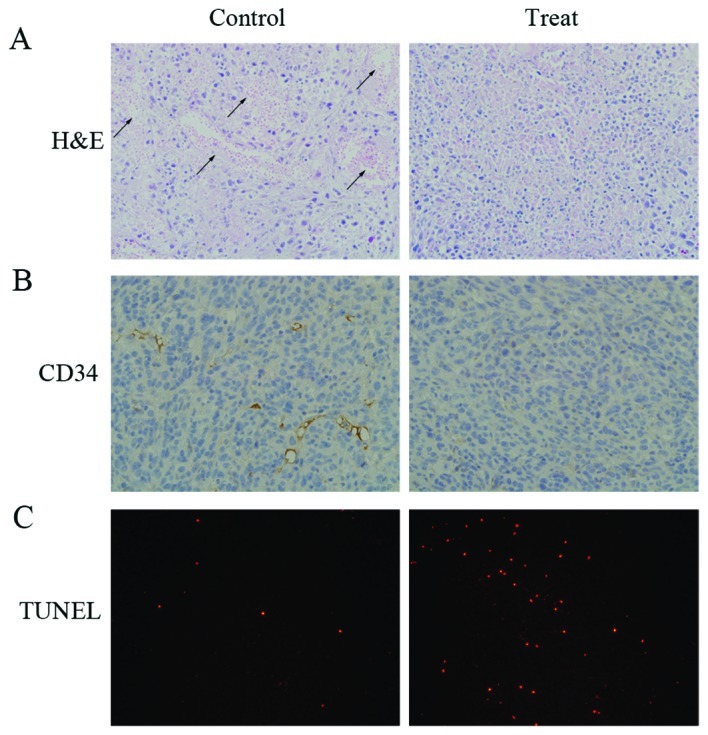
H&E, CD34 and TUNEL staining of tumor tissues. 4T1 xenograft tissues were removed and cut into 4-μm thick slices. Representative images of (A) H&E staining showing the necrotic areas, (B) CD34 stainig, as an indicator of tumor microvessel density, and (C) TUNEL staining, where TUNEL-positive cells indicate apoptosis. The sites of hemorrhagic necrosis are indicated by arrows.

To elucidate the mechanisms behind the effect of Huaier extract *in vivo*, the tumor tissues from the animal models were stained with H&E, CD34 and TUNEL (Fig. 7). The H&E-stained sections revealed large areas of necrosis occurring in both groups. The necrotic part in the untreated tumors was hemorrhagic. However, an ischemic type of necrosis with little or no blood was observed in the Huaier-treated tumors. We then performed TUNEL staining to explore the apoptotic effect induced by Huaier extract *in vivo*. The increased ratio of TUNEL-positive cells clearly demonstrated that the induction of apoptosis was involved in the antitumor activity of Huaier extract. Furthermore, CD34 staining was performed to evaluate the MVD in the tumor. Under microscopic analysis, a marked decrease in the expression of CD34 was observed in the treated group compared with the control group.

## Discussion

Conventional treatments for cancer patients include surgery, radiotherapy and chemotherapy. Recently, some alternative treatments, such as gene therapy and targeted therapy have attracted some attention. However, these therapies are usually unaffordable for most patients and have limited efficiency and serious side-effects. TCM has been used in China for thousands of years. Along with its anticancer effect, TCM has been widely applied to reduce toxic side-effects, improve quality of life, enhance immune function as well as prevent recurrence and metastasis for cancer patients (30). In recent years, TCM has been increasingly accepted and studied worldwide. For example, ‘Chong Lou Fu Fang’ was proven to improve the effect of chemotherapeutic agents on gastric cancer cells (31). Treatment with Iscador, extracted from mistletoe, resulted in a better survival among cancer patients (32). Despite of the ever-growing interest, rigorous and systematic pre-clinical evaluation is required for the globalization of TCM.

As an indispensable step for metastasis, angiogenesis is a promising target in anticancer therapy. Anti-angiogenic agents exert their effect in 2 ways (33,34). Direct inhibitors disrupt the proliferation, migration and differentiation of endothelial cells. On the other hand, indirect inhibitors interfere with the communication between tumor cells and endothelial cells by suppressing the expression of pro-angiogenic cytokines or blocking the binding of factors with their receptors. Although anti-angiogenic agents exhibit obvious antitumor activities, serious side-effects are often observed following treatment, such as hypertension, impaired wound healing, haemorrhaging and thrombosis (35). Therefore, novel natural herbs, such as grape seed extract (36) and dihydroartemisinin (DHA) (37), which have been proven to be safe for humans, are recognized as sources of effective antitumor agents.

Huaier, one of the most popular medical fungi in China, belongs to the Polyporaceae family and has been used as a TCM for almost 1,600 years. In the present study, the anti-angiogenic and antitumor effects of Huaier extract were assessed using HUVECs and 4T1 cells as a model. The results of MTT assay demonstrated that Huaier extract significantly attenuated the proliferation of HUVECs and 4T1 cells in a time- and dose-dependent manner (P<0.05). To our knowledge, this was the first study that investigated and demonstrated Huaier extract inhibited the proliferation of endothelial cells and mouse mammary tumor cells. Importantly, Huaier extract was more cytotoxic for the tumor cells with a lower IC_50_. In addition, the inhibition of the proliferation of HUVECs was caused by cell cycle arrest and pro-apoptotic activities. P21 is a well-studied cyclin-dependent kinase (CDK) inhibitor. It inhibits the activity of the cyclin-CDK2 or -CDK1 complex, leading to G1/S cell cycle arrest (38). After incubation with Huaier extract for 24 h, the protein level of p21 was increased. This suggested that Huaier extract caused cell cycle arrest in the HUVECs partly by promoting p21 accumulation.

In order to reveal the potential signaling pathways underlying the potent anti-angiogenic activity of Huaier extract, we investigated the expression of some angiogenic molecules. As one of the key pro-angiogenic molecules, VEGF is a highly specific mitogen for vascular endothelial cells and a potent vascular permeability enhancer. During the process of angiogenesis, VEGF is responsible for endothelial cell proliferation, migration, and antiapoptosis (39). Certain studies have demonstrated that the overexpression of VEGF in cancer patients is associated with a poor prognosis and decreased survival (40). As shown in Fig. 5, Huaier extract inhibited the expression of VEGF in a concentration-dependent manner. Among the upstream pathways that mediate VEGF expression, PI3K/AKT and MEK/ERK play important roles (41). As shown in Fig. 5, Huaier extract dose-dependently suppressed the activation of ERK without exerting any influence on AKT. Richard *et al*(42), as well as a previous study (43) demonstrated that active p42/p44 MAPK increased HIF-1-dependent transcriptional activity, which finally increased the expression of downstream proteins, including VEGF. Recently, the addition of U0126, a known selective inhibitor of MAPK/ERK kinase, was shown to inhibit the tube formation and induce apoptosis in HUVECs (44). Therefore, we hypothesized that Huaier extract could suppress the activation of ERK, inhibit VEGF expression and eventually exhibit anti-angiogenic activity. In addition, JNK, STAT3 and NF-κB are important pathways that regulate cell migration (45–47). In this study, we provide evidence that Huaier extract inhibits the phosphorylation of JNK, STAT3 and p65 (the major component in NF-κB complex). These data reveal the mechanisms underlying the anti-angiogenic activity of Huaier extract.

In addition to the anti-angiogenic and antitumor activities, the results from animal studies showed no significant adverse effects of Huaier extract on the body weights of the treated mice. As shown in Fig. 6E, in the treated group, gavage with 50 mg of Huaier extract per day did not cause body weight loss compared to the control group.

In conclusion, Huaier extract is a potent anti-angiogenic and antitumor agent. A gavage dose of 2.5 g/kg per day given to the mice was safe and effective against angiogenesis and solid tumor growth. These results highlight the possible application of Huaier extract in cancer chemoprevention and lay a solid foundation for clinical use in humans. However, further investigations are required to assess the detailed mechanisms, the responsible component(s) and to ascertain its beneficial role in the clinical setting.
